# Biochar-Based Remediation of Heavy Metal-Contaminated Soils: Mechanisms, Synergies, and Sustainable Prospects

**DOI:** 10.3390/nano15191487

**Published:** 2025-09-29

**Authors:** Yuxin Wei, Jingjing Ma, Kuankuan Liu, Shuai Zhang, Junqi Wang

**Affiliations:** Technology Innovation Center for Land Engineering and Human Settlements, Shaanxi Land Engineering Construction Group Co., Ltd. and Xi’an Jiaotong University, School of Human Settlements and Civil Engineering, Xi’an Jiaotong University, Xi’an 710049, China; weiyuxin5924@stu.xjtu.edu.cn (Y.W.); mjj20010218@stu.xjtu.edu.cn (J.M.); lk20010422@stu.xjtu.edu.cn (K.L.); zs0209@stu.xjtu.edu.cn (S.Z.)

**Keywords:** biochar, heavy metal pollution, soil remediation, adsorption mechanism, sustainable development

## Abstract

This study systematically explores the mechanisms and application potential of biochar in remediating heavy metal-contaminated soils. Particular emphasis is placed on the role of raw materials and pyrolysis conditions in modulating key physicochemical properties of biochar, including its aromatic structure, porosity, cation exchange capacity, and ash content, which collectively enhance heavy metal immobilization. The direct remediation mechanisms are categorized into six pathways: physical adsorption, electrostatic interactions, precipitation, ion exchange, organic functional group complexation, and redox reactions, with particular emphasis on the reduction in toxic Cr^6+^ and the oxidation of mobile As^3+^. In addition to direct interactions, biochar indirectly facilitates remediation by enhancing soil carbon sequestration, improving soil physicochemical characteristics, stimulating microbial activity, and promoting plant growth, thereby generating synergistic effects. The study evaluates combined remediation strategies integrating biochar with phytoremediation and microbial remediation, highlighting their enhanced efficiency. Moreover, practical challenges related to the long-term stability, ecological risks, and economic feasibility in field applications are critically analyzed. By synthesizing recent theoretical advancements and practical findings, this research provides a scientific foundation for optimizing biochar-based soil remediation technologies.

## 1. Introduction

### 1.1. Background and Scope

Soil heavy metal (HM) pollution is a significant environmental issue, primarily driven by anthropogenic activities such as mining, smelting, industrial emissions, transportation, and the use of pesticides and fertilizers [1,2]. Over the past five decades, global environments have accumulated substantial HM contaminants, with anthropogenic inputs exceeding 30 million tons of Cr and 80 million tons of Pb [3]. Soil contamination manifests as a global environmental challenge, particularly pronounced in industrialized nations. The European Union currently confronts remediation demands at over 500,000 contaminated industrial sites, while the United States has documented approximately 600,000 hectares of agricultural land rendered unproductive due to HM accumulation [2]. Developing countries exhibit equally concerning contamination patterns. In Lubumbashi, Democratic Republic of Congo, soil concentrations of Cu (1355 mg∙kg^−1^) and Cd (236 mg∙kg^−1^) significantly surpass WHO-recommended thresholds (Cu: 100 mg∙kg^−1^; Cd: 2 mg∙kg^−1^) [4]. Similarly, Kenya’s Migori gold mining region demonstrates severe polymetallic contamination, with maximum soil concentrations reaching 705.9 mg∙kg^−1^ Pb, 570 mg∙kg^−1^ Cd, 3591 mg∙kg^−1^ Zn, and 86.3 mg∙kg^−1^ Ni—all exceeding maximum allowable concentrations (MACs) for agricultural soils [5]. China’s rapid industrialization and urbanization have engendered distinctive contamination characteristics: a high total load, elevated concentrations, and complex contamination profiles. The 2014 National Soil Pollution Status Survey Bulletin revealed 16.1% of surveyed sites exceeding contaminant thresholds, with inorganic pollutants (notably Cd and As) contributing to 82.8% of cases. Mining-affected areas span 2 million hectares, significantly surpassing global averages.

The accumulation of HMs in soil, including Cd, Pb, and As, poses multifaceted threats to ecosystems and human health. These persistent contaminants impair soil microbial diversity and enzymatic functions, disrupting nutrient cycling and organic matter decomposition, which ultimately degrades soil fertility and agricultural productivity [6]. Furthermore, HM ions can migrate into groundwater or adsorb onto airborne particulates, amplifying ecological risks through cross-media contamination. Human exposure occurs via inhalation, dermal contact, and the consumption of contaminated crops, with Cd and Pb specifically implicated in renal dysfunction, cardiovascular diseases, and neurodevelopmental disorders. Their carcinogenic potential, particularly for Cr and As, exacerbates long-term public health burdens [7]. The food chain further magnifies these risks, as bioaccumulation in crops and biomagnification in livestock intensify human intake [8]. As a result, the remediation of HM-contaminated soils has become a critical environmental challenge.

Currently, soil remediation methods are generally categorized into physical, chemical, and biological approaches. Physical remediation is often time-consuming, expensive, and of limited applicability [9]. Phytoremediation leverages hyperaccumulator plants for cost-effective and non-invasive soil cleanup while preserving agricultural utility. However, its slow metal extraction rate, limited root-depth coverage, and inefficiency in multi-contaminant systems hinder practical implementation [10]. Microbial remediation utilizes metal-resistant strains for pollutant transformation or immobilization but requires strict control of pH, organic matter, and temperature for optimal activity [11]. Both methods face toxicity challenges: phytoremediation risks secondary contamination through plant biomass disposal, while microbial viability declines under high metal stress, necessitating costly bioaugmentation. Chemical methods are categorized into chemical leaching and chemical passivation. Chemical leaching employs acids or chelators to mobilize metals but risks secondary pollution via reagent residues [12]. In contrast, chemical passivation stabilizes metals through low-cost amendments (e.g., biochar, phosphates), offering simplicity and immediate effectiveness [13]. Among passivation materials, biochar is notable for its availability, simple preparation, and high adsorption capacity, making it a promising candidate for large-scale application [14]. Recent studies have increasingly focused on the mechanisms of heavy metal immobilization by biochar, as well as its synergistic applications with plant and microbial remediation. To provide a comprehensive and up-to-date synthesis of these developments, this review analyzes relevant literature published between 2013 and 2025. The methodology used to conduct the literature review, including the database selection, keyword strategy, and screening criteria, is described in Section 1.2. From the dataset, 111 relevant articles were selected for in-depth review, focusing on the primary mechanisms by which biochar immobilizes HMs, including physical adsorption, electrostatic adsorption, precipitation, ion exchange, complexation with organic functional groups, and redox reactions. The study further analyzes the synergistic mechanisms of biochar–plant/microbial combined remediation systems. Based on evaluations of long-term stability, ecological risks, and economic feasibility in field applications, it identifies core challenges in biochar material design, including stability and selectivity regulation, as well as trade-offs in environmental benefits. These findings provide a theoretical foundation for optimizing biochar-based remediation materials and supports the large-scale application of biochar in soil remediation. Compared with previous reviews, this manuscript provides a more integrated framework by categorizing six distinct remediation mechanisms of biochar and emphasizing its synergistic role with plants and microorganisms. It also links biochar’s physicochemical properties to its remediation performance and critically evaluates long-term stability and field applicability. The objective of this review is to synthesize recent advances in the biochar-based remediation of heavy metal-contaminated soils, clarify underlying mechanisms, and identify key challenges for sustainable application.

### 1.2. Methodology of the Literature Review

**Figure 1:**
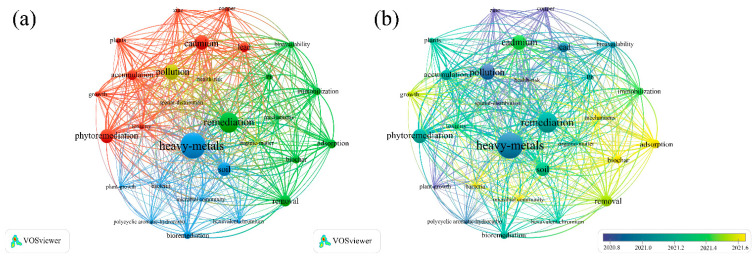
(**a**) Cluster visualization map generated by VOSviewer v1.6.20 using the keywords “HM contamination” and “soil remediation”. (**b**) Overlay visualization map (timeline view) generated by VOSviewer v1.6.20 using the keywords “HM contamination” and “soil remediation”.

To identify current research trends in the biochar-based remediation of heavy metal-contaminated soils, we first conducted a comprehensive literature search using the Web of Science Core Collection. The keywords “heavy metal contamination” and “soil remediation” were used to retrieve relevant publications from 2013 to 2025, resulting in an initial dataset of 3198 articles. Keyword co-occurrence and overlay visualization maps were generated using VOS viewer v1.6.20, as shown in Figure 1. The results indicate that recent research has increasingly focused on the mechanisms of heavy metal adsorption by biochar, as well as the integration of biochar with plant-based and microbial remediation techniques. These trends reflect a growing interest in multi-mechanistic and synergistic approaches to soil remediation.

Based on these insights, we further screened titles, abstracts, and full texts to identify studies specifically addressing biochar-based remediation mechanisms and synergistic strategies. A total of 111 articles were selected for in-depth analysis. The inclusion criteria focused on studies that investigated (1) the physicochemical properties of biochar relevant to heavy metal immobilization, (2) direct remediation mechanisms such as adsorption, ion exchange, and redox reactions, and (3) synergistic applications involving plants or microorganisms. The selected literature was categorized based on biochar preparation methods, feedstock types, pyrolysis conditions, and the specific heavy metals targeted. This structured approach enabled a detailed synthesis of mechanisms, material design strategies, and synergistic remediation systems, forming the foundation of this review.

## 2. Preparation of Biochar

### 2.1. Preparation Methods

The most common method for preparing biochar is pyrolysis, which typically occurs at temperatures between 450 °C and 550 °C, where biomass is rapidly heated to produce biochar. However, due to the rapid heating rate and high reaction temperatures during pyrolysis, the resulting biochar often contains many unstable components and has a relatively low solid yield of about 10% [15]. To increase the yield of biochar and achieve more thorough the decomposition of the raw materials [16], researchers often control the pyrolysis temperature range between 200 °C and 800 °C and adopt a slower heating rate (0.1–1.0 °C∙s^−1^) [17]. Under these conditions, the solid yield of biochar can typically reach 50% or higher [18].

Considering the characteristics of different raw materials and application needs, hydrothermal carbonization has been increasingly applied to convert wet biomass waste into biochar. This method operates at relatively low temperatures (180–250 °C) and high pressures (2–10 MPa) over an extended reaction time (8–10 h), producing biochar rich in oxygen-containing functional groups with a high cation exchange capacity (CEC), which enhances its adsorption performance in soil remediation [15]. However, biochar derived from hydrothermal carbonization exhibits weaker electrostatic adsorption and electron transfer capabilities, and the prolonged reaction time limits its practical application. To address these limitations, alternative methods such as microwave carbonization and gasification have been explored. Microwave carbonization enables rapid and uniform heating, significantly reducing the reaction time while allowing for precise process control [19]. Despite its efficiency, the biochar produced still requires further improvements in stability and quality. Gasification, a relatively simple and cost-effective approach, offers fast biochar production. However, it yields lower biochar quantities and results in heterogeneous particle sizes, which may limit its effectiveness in specific applications. By selecting appropriate biochar preparation techniques, key physicochemical properties, including pore structure, specific surface area (SSA), and functional group composition, can be optimized to meet the demands of various pollution remediation scenarios.

### 2.2. Effects of Raw Materials and Pyrolysis Temperature on the Physicochemical Properties of Biochar

During pyrolysis, the temperature and raw material type are the primary factors influencing biochar’s physicochemical properties and its capacity to adsorb and immobilize HMs in soil. To gain a clearer understanding of biochar’s role in remediating HM-contaminated soils, Table 1 presents a summary of its key physicochemical properties under various pyrolysis conditions, including its aromaticity, ash content, pore structure, CEC, and pH.

As quantitatively demonstrated in Table 1, elevated pyrolysis temperatures enhance biochar’s ash content, aromaticity, and porosity. However, excessive heating induces volatile matter release, leading to pore blockage and a reduced surface area [26]. Such structural modifications directly determine the SSA and availability of adsorption sites, thereby influencing biochar’s HM adsorption capacity. Notably, the pyrolysis temperature not only alters physical structures but also significantly modifies biochar’s pH characteristics through mineral component transformation (e.g., carbonate formation) and surface functional group reorganization [27]. Moreover, as the temperature increases, alkaline salts (such as carbonates) are progressively released, leading to a rise in pH. Above 300 °C, this effect intensifies, and by approximately 600 °C, the pH stabilizes [28].

In addition to the preparation temperature, the types of raw materials also co-regulate the pore structure and surface chemical properties of biochar through their own composition differences (e.g., mineral elements, ash content) and ultimately affect its environmental application efficiency. The feedstock type also plays a critical role in determining the cation exchange capacity (CEC) of biochar, which directly influences its ion exchange efficiency. Trakal et al. [29] systematically compared the Cd and Pb adsorption capacities of wheat straw biochar and grapevine stem biochar under identical experimental conditions. Notably, despite sharing comparable SSA values (70 mg^2^·g^−1^), wheat straw biochar exhibited a 2.14-fold higher CEC (402 mmol·kg^−1^) than grapevine stem biochar, highlighting the substantial impact of feedstock selection on material functionality. Biochar can be derived from a wide range of raw materials, including plant-based, animal-based, and sludge-based sources. Plant-based biochar typically has a high SSA, a rich pore structure, and various oxygen-containing functional groups. These characteristics enable it to effectively remove certain HMs, such as Pb and Cu, with removal efficiencies ranging from 81% to 95% [30,31]. However, due to the complex forms of As and Cd in the soil and their weak binding interactions with the functional groups on the biochar surface, plant-based biochar tends to exhibit relatively low removal efficiencies for these metals, generally around 30% [32,33]. In contrast, animal-based biochar (e.g., manure-derived biochar) contains more nitrogen, minerals (Ca, Mg, Fe), and functional groups (carboxyl [-COOH], carbonyl groups [C=O]), leading to higher aromaticity, ash content, and CEC [34]. The mineral components within high-ash biochars chemically immobilize HMs through precipitation reactions, effectively reducing bioavailability while demonstrating notable removal capacities for Cr^3+^ and Ni^2+^. Its high CEC also facilitates the adsorption and immobilization of metal cations such as Pb^2+^ and Cd^2+^ [35]. Furthermore, sludge-derived biochar, which is rich in mineral elements and has a high CEC, not only increases the content of dissolved organic matter in the soil, thereby reducing the mobility of As [36], but also provides additional binding sites for HM ions, enhancing the immobilization of As. However, sludge biochar itself contains HMs, which poses a risk of desorption and potential release back into the environment [37].

## 3. Mechanisms of HM Remediation by Biochar

**Figure 2:**
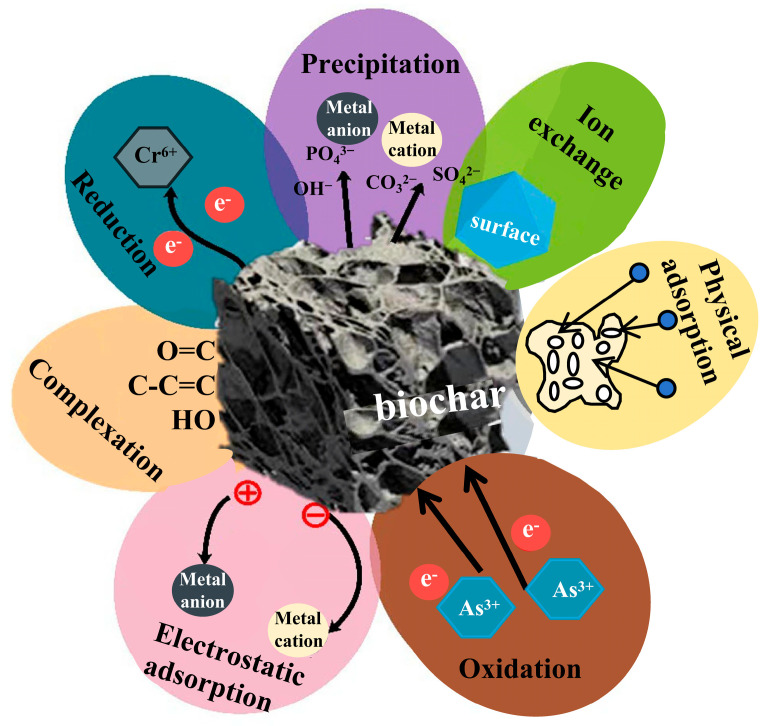
Mechanism of HM adsorption by biochar materials (redraw according to literature [38]).

The process of remediating HM-contaminated soil using biochar involves multiple mechanisms, including physical adsorption, electrostatic adsorption, co-precipitation, complexation reactions, and redox reactions (Figure 2). The synergistic interactions among these mechanisms determine the effectiveness of biochar in immobilizing HM ions. The following sections will detail the specific principles of these immobilization mechanisms and their performance in HM removal.

### 3.1. Physical Adsorption

**Figure 3:**
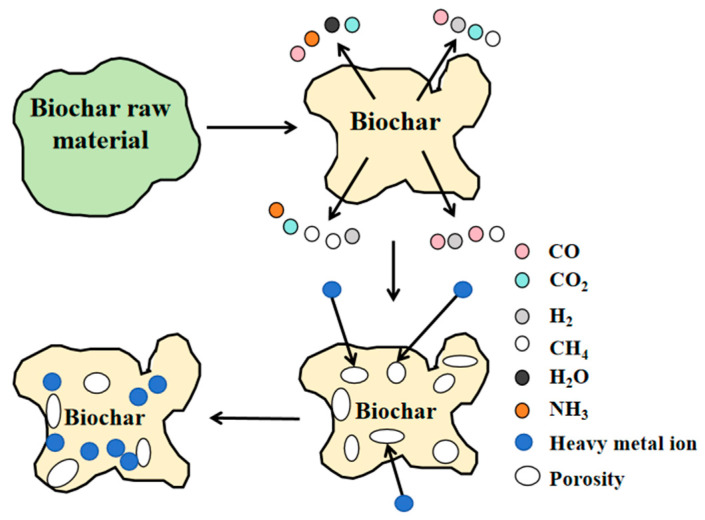
Physical adsorption mechanism of biochar (redraw according to literature [40,41]).

Physical adsorption serves as a fundamental mechanism in the remediation of HM-contaminated soils. This process primarily depends on the porous structure and large SSA of biochar, which facilitates the adsorption of HM ions via Van der Waals forces [39]. Unlike chemical adsorption, physical adsorption does not involve bond formation but instead retains HMs through weak intermolecular interactions. The well-developed pore structure of biochar provides abundant adsorption sites, effectively capturing and immobilizing HM ions in the soil (Figure 3). Moreover, its high SSA enhances contact with contaminants, significantly improving adsorption efficiency. Notably, physical adsorption, primarily governed by micropore filling and van der Waals interactions, tends to remain stable under near-neutral to mildly alkaline conditions. Unlike electrostatic adsorption, it does not depend on surface charge modulation. However, in strongly acidic soils (pH below 5), the expansion of hydration shells and competition with protons can impede the diffusion of metal ions into micropores, thereby reducing adsorption efficiency. As such, physical adsorption should be distinguished from pH-sensitive electrostatic mechanisms, and its effectiveness is constrained by specific soil chemical conditions.

The physical adsorption capacity of unmodified biochar for HMs is inherently limited by its underdeveloped pore structure and low surface reactivity. For instance, biochar derived from bagasse under low pyrolysis temperatures (200–300 °C) exhibits a SSA of merely 3.9–10.7 m^2^·g^−1^, resulting in suboptimal Pb^2+^ adsorption rates below 20% [42]. These constraints highlight the necessity of chemical modifications to enhance biochar’s structural and adsorptive properties. Metal-based modifications, particularly through oxide nanoparticle integration, have proven effective in creating hierarchical pore architectures. A representative study demonstrated that MgO-functionalized biochar achieved a 380-fold increase in SSA (from 0.07 to 26.56 m^2^·g^−1^) and tripled Pb^2+^ removal efficiency (from 23% to 74%) compared to pristine biochar [43]. This enhancement mechanism arises from two synergistic effects: (1) MgO nanoclusters providing additional binding sites through surface complexation, and (2) the expanded pore network facilitating the intraparticle diffusion of metal ions.

Beyond the preparation of metal–biochar composites, various modification techniques, such as SC(NH_2_)_2_ impregnation, H_2_O_2_ oxidation, and KOH activation, can significantly alter the surface properties of biochar (Table 2). However, HM immobilization by biochar is governed by multiple synergistic mechanisms, making the relationship between surface modifications and immobilization efficiency non-linear. For instance, studies by Song et al. [44] and Fan, Cai, Chi, Reid, Coulon, Zhang and Hou [8] reported that although modification reduced the SSA of biochar, the strong binding affinity of manganese oxides (MnO*_x_*) for Cu^2+^ and the complexation of thiol groups (R-SH) with Cd^2+^ enhanced HM immobilization. In contrast, certain modifications that increase the SSA may not necessarily improve immobilization efficiency. For example, Liu et al. [45] found that acid treatment reduced the aromaticity of biochar, disrupted its π-conjugated aromatic structure, and weakened cation–π interactions, thereby hindering the formation of stable complexes with Zn^2+^.

### 3.2. Electrostatic Adsorption

**Figure 4:**
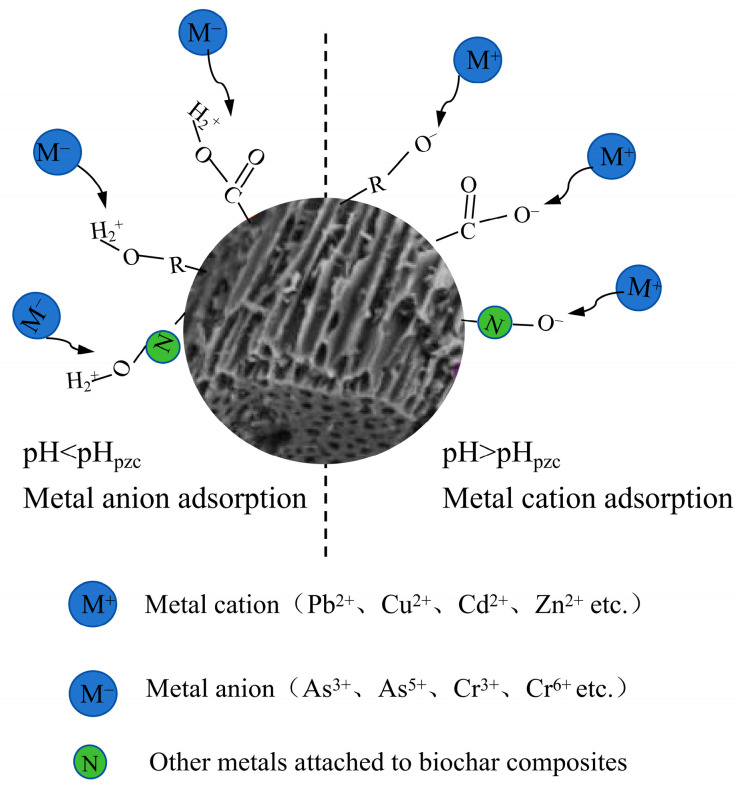
Mechanism of electrostatic adsorption of HMs in soil by biochar materials (redraw according to literature [49,50]).

The effectiveness of physical adsorption is inherently limited, particularly when biochar exhibits a low surface area and pore volume. To compensate for this limitation, electrostatic adsorption plays a crucial role when biochar surfaces carry a charge (Figure 4). Biochar typically contains various functional groups (e.g., -COOH, phenolic hydroxyl group (Ar-OH)), which dissociate under specific pH conditions, imparting a net negative charge to the surface. This charge facilitates the electrostatic attraction of positively charged HM ions in the soil, thereby enhancing their immobilization on the biochar surface [48]. The efficiency of electrostatic adsorption is governed by multiple factors, including soil pH, ionic strength, biochar surface charge density, and the nature of the functional groups present. At higher pH levels, functional groups such as -COOH and Ar-OH undergo increased dissociation, intensifying the negative charge on the biochar surface and thereby strengthening electrostatic interactions with HM cations [27]. Additionally, a higher CEC enables biochar to attract and retain more positively charged metal ions, further improving its immobilization performance in contaminated soils.

**Figure 5:**
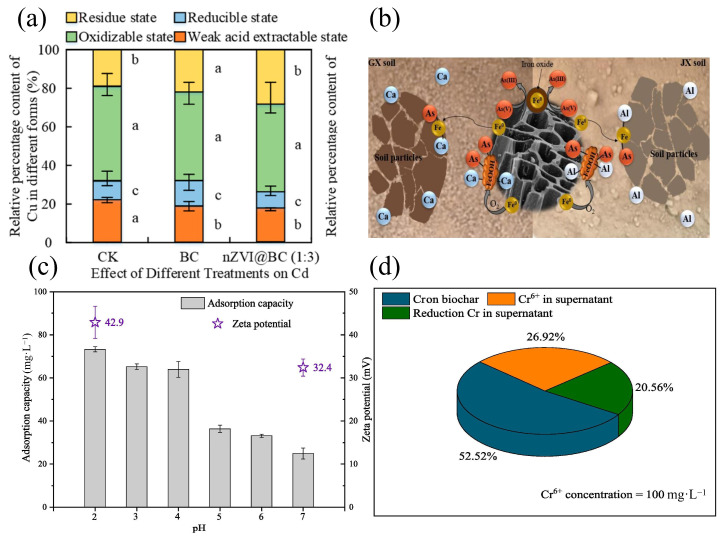
(**a**) Effects on Cd speciation under different treatments after 40 days of incubation. Note: Those with the same letters in each group indicated that the difference was not significant (*p* > 0.05), while those with different letters indicated that the difference was significant (*p* < 0.05) [51]. (**b**) One-pot synthesized nZVI/BC effectively immobilizes arsenic [52]. (**c**) Effects of pH on Cr^6+^ removal by the optimal Fe@Zn@HBC [53]. (**d**) The chromium distribution after saturated adsorption in solution and biochar [53].

The surface charge modification of biochar significantly enhances its electrostatic immobilization capacity for HMs through pH-dependent ion exchange mechanisms. For instance, Duan et al. [51] demonstrated that nZVI@BC composites release alkaline ions (K^+^, Na^+^, Ca^2+^, Mg^2+^), which replace exchangeable H^+^ and Al^3+^ in acidic soils (pH < 5.5). This ion displacement increased soil pH, collectively enhancing the Cd^2+^ adsorption capacity by 3.22-fold compared to pristine biochar, as shown in Figure 5a. The pH-regulated charge reversal mechanism was further validated by Fan et al. [52] using nZVI/BC composites (pH_pzc_ = 8). When applied to near-neutral soils (pH = 7.9), the biochar surface maintained negative charges (pH < pH_pzc_), enabling more than 90% As immobilization through the electrostatic attraction of HAsO_4_^2−^ species, as shown in Figure 5b. This dual functionality—alkaline ion exchange for cationic metals (e.g., Cd^2+^) and pH-switchable adsorption for oxyanions (e.g., As^5+^)—reveals the multi-mechanistic synergy in biochar-based remediation.

The pH-driven electrostatic mechanism universally governs HM immobilization across varied species. Extending the pH-regulated electrostatic adsorption principles demonstrated for Cd^2+^ [51] and As^5+^ [52], Yu et al. [53] explicitly quantified the electrostatic dominance in Cr^6+^ immobilization by Fe@Zn@HBC biochar. Their Cr speciation analysis revealed that 52.52% of total Cr^6+^ removal under acidic conditions (pH < 4) originated from direct adsorption onto protonated biochar surfaces, as shown in Figure 5c,d. This electrostatic priority aligns with the alkaline-driven Cd^2+^ adsorption enhancement (3.22-fold efficiency gain [51]), collectively establishing surface charge modulation as the universal trigger for HM sequestration. While subsequent Cr^6+^ reduction and precipitation occurred, the adsorbed Cr^3+^ retention rate remained strictly dependent on the initial electrostatic capture efficiency [53], echoing the multi-stage immobilization patterns observed in Cu^2+^, Zn^2+^, and Pb^2+^ systems [54,55].

### 3.3. Precipitation Reaction

**Figure 6:**
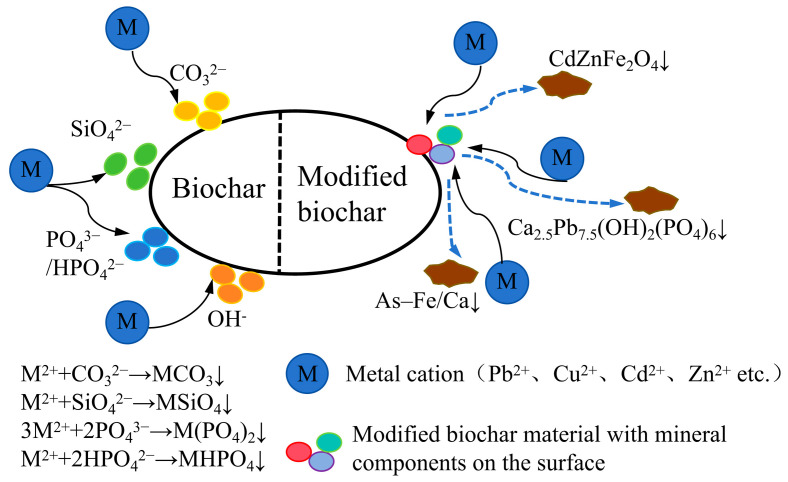
Mechanism of precipitation reaction of HMs with biochar materials.

Co-precipitation is a crucial mechanism through which biochar facilitates HM remediation in soil. This process involves the formation of insoluble or sparingly soluble metal precipitates, effectively immobilizing HMs from the soil solution (Figure 6). Due to their extremely low solubility, these precipitates remain stable over time, minimizing the risk of HM re-release into the soil and thus ensuring long-term stabilization. Precipitation mechanisms include two main types: (1) co-precipitation with mineral anions such as carbonates and phosphates released from high-ash biochars and (2) surface nucleation of metal hydroxides or carbonates directly on biochar surfaces. While these precipitates are generally stable under neutral to alkaline conditions, partial remobilization may occur under acid pulses, CO_2_ degassing, or organic acid exudates. For example, PbCO_3_ and CdCO_3_ can dissolve under pH < 5 or in the presence of chelating agents, affecting long-term stability. Moreover, the coprecipitation efficacy of biochar is primarily governed by three interdependent factors: mineral composition (determined by pyrolysis feedstocks), pyrolysis temperature, and ambient pH regulation. As systematically documented in Table 3, these parameters collectively dictated the effectiveness of HM immobilization through coprecipitation mechanisms in recent experimental studies, providing critical benchmarks for process optimization.

As demonstrated in Table 3, most HM ions in contaminated soils can react with anions such as OH^−^ CO_3_^2−^, and PO_4_^3−^ to form insoluble precipitates. A typical example is Pb^2+^, which reacts with these anions to form Pb(OH)_2_, PbCO_3_, Pb_3_(CO_3_)_2_(OH)_2_, and Pb_3_(PO_4_)_2_, thereby immobilizing lead in the soil [62]. For instance, Ahmad, Ok, Kim, Ahn, Lee, Zhang, Moon, Al-Wabel, and Lee [31] demonstrated that applying 10% soybean straw biochar and pine needle biochar to soil effectively promoted the formation of Pb_5_[PO_4_]_3_OH and Pb_5_[PO_4_]_3_Cl precipitates, significantly reducing Pb mobility and achieving a removal efficiency of 64.31–95%. Additionally, biochar derived from poultry manure and sludge, characterized by its high ash content, is rich in phosphates, which significantly enhance the precipitation and immobilization of Pb^2+^ [62]. Park et al. [63] demonstrated that biochar derived from poultry manure and sludge, due to its high ash content and abundant phosphates, significantly enhances Pb^2+^ precipitation, achieving an exceptional immobilization rate of 99.99%. In contrast, under identical conditions, woody biochar exhibited a lower fixation rate of 72.9%. The efficiency of Pb immobilization is influenced not only by biochar type but also by soil pH. For instance, Igalavithana et al. [64] observed that in acidic soil (pH = 4.9), the application of 5% pinecone biochar resulted in a −20% fixation rate for Pb, indicating that acidic conditions dissolved Pb precipitates, thereby diminishing immobilization efficiency. This underscores the need for further biochar modification under acidic conditions to enhance its stability and effectiveness.

The mineral components in biochar not only effectively precipitate and immobilize Pb^2+^ but also combine with Cu^2+^ to form Cu(OH)_2_ and CuCO_3_ [65]; with Cd^2+^ to produce Cd_3_(PO_4_)_2_ and CdCO_3_ [65]; and with AsO_4_^3−^ to generate (Fe/Ca)AsO_3_∙SiO_3_ and (Fe/Ca)AsO_4_∙SiO_3_ precipitates [52], thereby achieving immobilization. Furthermore, the mineral content is closely associated with the pyrolysis temperature. High-temperature biochar (≥500 °C) releases significantly more CO_3_^2−^, approximately an order of magnitude higher than low-temperature biochar (≤400 °C), making precipitation reactions predominant under such conditions [66].

### 3.4. Ion Exchange

**Figure 7:**
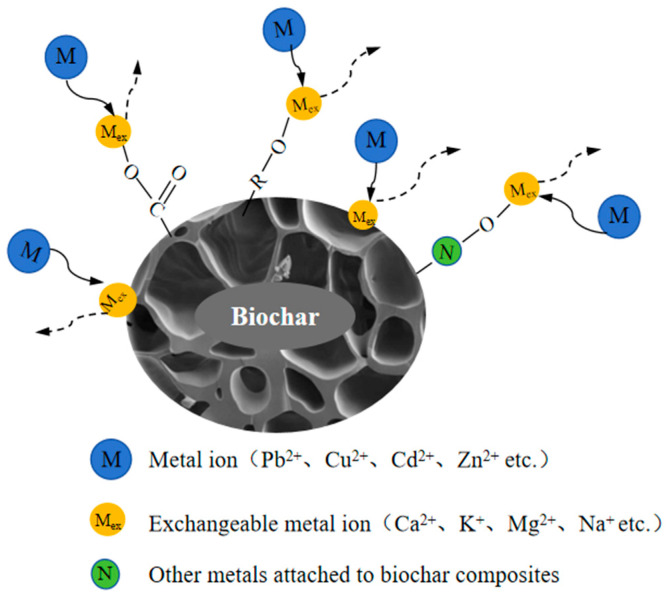
Mechanism diagram of ion exchange between biochar and HM ions (redraw according to literature [50,67]).

In soil environments, ion exchange primarily occurs through the dissociation of negatively charged functional groups (e.g., -COOH, -OH, Ar-OH) on the biochar surface under specific conditions. This process releases H^+^ or other cations (e.g., K^+^, Ca^2+^, Na^+^, Mg^2+^), which subsequently exchange with HM ions (e.g., Pb^2+^, Cu^2+^ Cd^2+^). As a result, HM ions are adsorbed onto the biochar surface, while less harmful metal ions are released into the soil (Figure 7).

**Figure 8:**
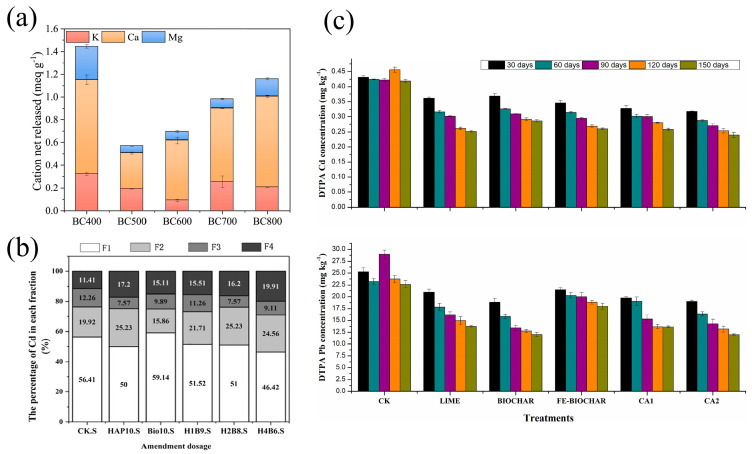
(**a**) Net release of AAEMs after adsorption [68]. (**b**) The chemical distribution of HMs in the samples was estimated by the sequential chemical extraction procedures [69]. (**c**) Effect of soil additives on available Cd and Pb in incubated soil with time changes [70].

Jiang et al. [68] investigated the mechanism of Pb^2+^ immobilization using biochar derived from soybean straw and found that Ca^2+^ plays a predominant role in ion exchange, with exchange rates ranging from 55.3% to 75.6%, as shown in Figure 8a. This is primarily due to the identical valence state of Ca^2+^ and Pb^2+^ (in contrast to K^+^ and Na^+^) and the closer ionic radius of Ca^2+^ to Pb^2+^ (compared to Mg^2+^), facilitating the retention of Pb^2+^ in biochar while Ca^2+^ is released. To further enhance the efficiency of HM immobilization in soil, researchers have developed various biochar modification strategies. For example, Chen et al. [69] employed hydroxyapatite/calcium silicate hydrate modifications, while Hamid et al. [70] incorporated Ca^2+^-containing additives such as lime, significantly increasing the Ca^2+^ content in biochar, as shown in Figure 8b,c. These modifications enhance the ion exchange capacity, thereby improving the immobilization efficiency of HMs.

The raw material source plays a critical role in determining the CEC of biochar, thereby directly influencing its ion exchange efficiency.

### 3.5. Organic Functional Group Complexation

**Figure 9:**
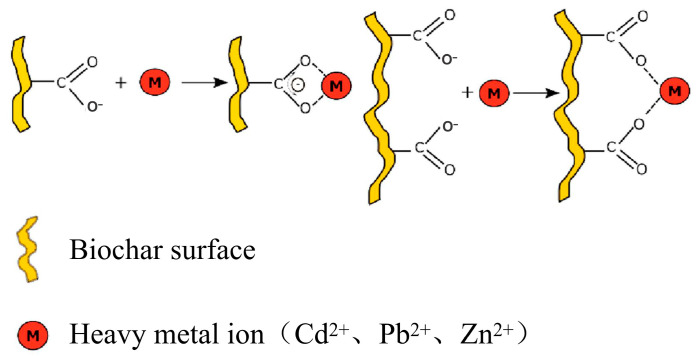
Complexation mechanism of HMs with biochar materials [71].

The organic functional groups on biochar surfaces play a crucial role in the complexation of HMs through various coordination mechanisms (Figure 9). As shown in Table 4, almost all HM ions in contaminated soils can undergo complexation reactions with oxygen-containing functional groups on the surface of biochar, particularly -OH and -COOH. Such reactions form stable complexes through coordination bonds between these functional groups and HM ions, thereby reducing the mobility and bioavailability of HMs.

Oxygen-containing groups, such as -COOH and -Ar-OH, primarily facilitate cation exchange, making them particularly effective for the immobilization of Pb^2+^ and Cd^2+^, especially under neutral to alkaline conditions. Moreover, nitrogenous moieties in biochar exhibit distinctive coordination capabilities, such as amino (-NH_2_) and pyridinic-N, exhibit pH-dependent behaviors, shifting from cation coordination to anion adsorption through protonation–deprotonation equilibria. The synthesis conditions also influence the preservation of these functional groups, with pyrolysis temperatures between 300 and 500 °C being optimal for retaining oxygen groups, while temperatures above 600 °C promote nitrogen enrichment through Maillard-derived reactions [77]. Additionally, the type of feedstock, whether lignocellulosic or manure-based, affects the inherent functional group density and post-synthesis modifications. Furthermore, soil pH plays a critical role in modulating complexation efficiency, with oxygen groups generally being more effective at a pH > 6, while nitrogen groups dominate in acidic conditions. This pH-dependent behavior aligns with the hard–soft acid-base theory [78].

While oxygen-containing functional groups (e.g., -COOH, hydroxyl [-OH]) are critical for biochar’s HM immobilization capacity, their thermal instability poses significant limitations. The pyrolysis-induced decomposition of lignocellulosic components (>300 °C for cellulose, 500–700 °C for lignin) results in 80–95% depletion of surface oxygen groups at elevated temperatures, directly compromising metal complexation efficiency. This inherent constraint necessitates strategic modifications to enhance biochar’s environmental remediation potential.

Metal oxide engineering has emerged as a promising modification approach. Iron oxides (Fe*_x_*O*_y_*) and manganese oxides (MnO*_x_*) synergistically enhance biochar’s complexation capacity through two mechanisms: (1) surface charge modulation via -OH group enrichment and (2) the formation of ternary metal–oxide–biochar complexes. Zhou et al. [79] demonstrated this dual enhancement through Fe-Mn oxide modified biochar, which achieved superior adsorption capacities for Cu^2+^ (64.9 mg·g^−1^) and Cd^2+^ (101.0 mg·g^−1^)—representing 3-fold and 3.6-fold improvements over pristine biochar, respectively. The enhanced performance stems from stable coordination structures (-COO-M and Fe-Mn-O-M bridges) confirmed through X-ray photoelectron spectroscopy analysis. Complementary studies by Gong et al. [80] revealed that Fe-O moieties facilitate ligand exchange reactions with Pb^2+^ while surface hydroxyls participate in inner-sphere complexation. This modification strategy increases active site density by 40–60% compared to unmodified biochar, effectively compensating for thermal degradation losses of native functional groups [48]. The strategic integration of multi-valent metal oxides not only overcomes the pH-dependent limitation of single-component systems but also establishes redox-active interfaces for persistent contaminant sequestration. Future optimization should focus on oxide phase stability under extreme soil conditions while leveraging waste-derived metal sources to ensure scalable sustainability.

### 3.6. Reduction-Oxidation Reaction

The immobilization of HMs in contaminated soils requires redox-mediated stabilization for certain toxic species with high mobility. Unlike direct adsorption mechanisms, redox-sensitive metals (e.g., Cr^6+^, As^3+^) require valence state transformation to less mobile forms (Cr^3+^, As^5+^) prior to immobilization. Biochar achieves this through two redox-active components: (1) oxygenated functional groups (quinones, phenolic [-OH]) serving as electron mediators, and (2) conjugated π-electron systems in graphitic domains with electron-donating/accepting capacities [81]. Spectroscopic evidence reveals that C=O facilitates Cr^6+^ reduction through single-electron transfer mechanisms, while persistent free radicals in biochar’s aromatic matrix enable As^3+^ oxidation via hydroxyl radical (·OH) generation. This dual redox functionality allows for simultaneous metal (loid) transformation (75–92% efficiency) and stabilized species complexation [82]. The following subsections delineate the oxidative transformation and reductive immobilization pathways, with mechanistic insights into their respective driving forces.

#### 3.6.1. Oxidation Reaction

In the oxidation mechanism, biochar reacts with HMs through its surface functional groups (e.g., -OH and C=O), accepting electrons from the HMs and oxidizing them from a lower to a higher valence state. For example, in the case of As, biochar can oxidize As^3+^ to As^5+^. As^5+^ is more stable and less toxic than As^3+^ in the environment and is more easily immobilized by other substances (e.g., Fe_x_O_y_ and aluminum oxides (Al_2_O_3_)) in the soil. The reaction equation is shown as Equation (1):

(1) $$H_{3}AsO_{3}-2e^{-}+H_{2}O\to H_{3}AsO_{4}+2H^{+}$$

**Figure 10:**
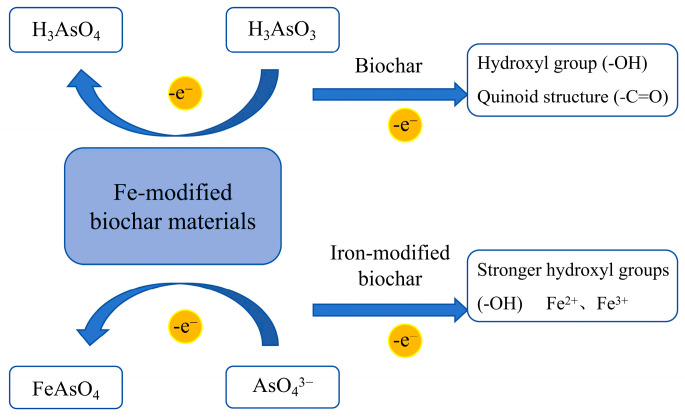
Oxidation mechanism of As^3+^ by Fe-modified biochar materials (the diagram was adapted from the layout of the reduction mechanism figure in the literature [87]).

Studies have shown (as illustrated in Table 5) that most raw biochar has a poor immobilization effect on As and may even exhibit adverse effects [83,84]. The reason is that when biochar is applied to soil, it increases soil pH and releases dissolved organic carbon and its negatively charged surface functional groups limit the adsorption of As [85]. To address this issue, raw biochar needs to be modified, with Fe being one of the most commonly used modifiers due to its high affinity for As. Typically, biochar is modified by adding ferric chloride (FeCl_3_), ferrous sulfate (FeSO_4_), magnetite (Fe_3_O_4_), hematite (Fe_2_O_3_), or zero-valent iron (Fe^0^) [55]. This modification increases the content of Fe*_x_*O*_y_*, enabling the biochar surface to contain more Fe-OH groups. These groups can adsorb and oxidize As^3+^ and As^5+^, thereby significantly improving the efficiency of As immobilization, as shown in Figure 10. In addition to Fe, MnO*_x_* is also used as a modifier. Biochar modified with MnO_x_ possesses a stronger positive charge on its surface, enabling it to attract As^3+^ more effectively and oxidize it into the more stable As^5+^. Further studies have shown that compared to using Fe or MnO_x_ alone, Fe-Mn binary mixed-modified biochar exhibits a higher SSA and enhances the oxidation of As^3+^ to As^5+^ in the outer sphere, resulting in greater adsorption capacity [86].

**Table 5:** Immobilization of HM As by biochar and composite materials.

Raw Materials	Pyrolysis Temperature (°C)	Biochar pH Value	Modification Method	Soil pH Value	Biochar Addition	HM Immobilization Effect	Ref
Eggshell–corncob	450	9.66	/	5.56	2%	−53.4%	[84]
Eggshell	450	9.53	/	5.56	2%	−40.1%	
Corncob	450	9.83	/	5.56	2%	−67.8%	
Rice straw	400	8.32	/	4.65	2%	−26%	[83]
Peanut shells	300	7.6	/	8.00	10%	−112%	[88]
Pine needles	700	9.7	/	8.00	10%	−32%	
Rice straw	300	/	FeCl_3_	7.50	1%	25%	[89]
Rice straw	300	/	Fe^0^	7.50	1%	35%	[89]
Eggshell–corncob	450	8.7	FeSO_4_·7H_2_O	5.56	2%	56%	[90]
Red cedar sawdust	300	9.7–10.3	Fe_3_O_4_	5.7	9%	20–30%	[91]
Sawdust	800	8.3	Fe_2_O_3_	8	2–5%	93.7–97.7%	[52]
Corn stalks	600	10.8	KMnSO_4_	7.21	1%	58%	[32]

#### 3.6.2. Reduction Reaction

**Figure 11:**
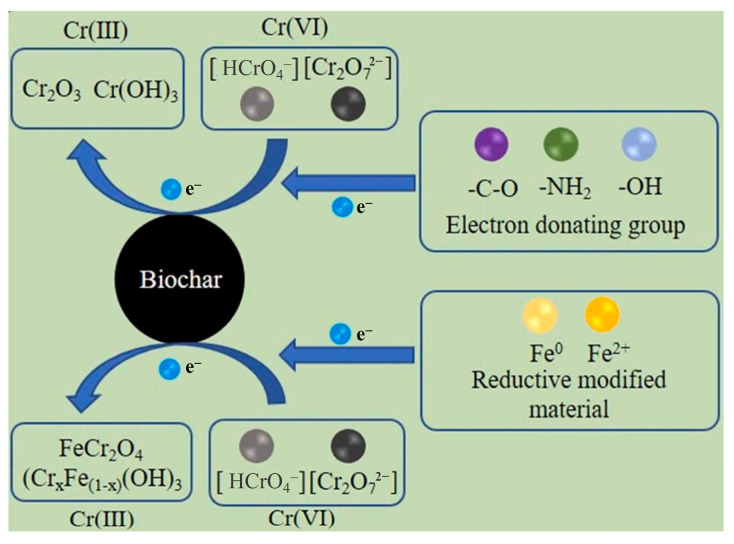
Reduction mechanism of Cr^6+^ by biochar materials [87].

Biochar acts as a reducing agent during the reduction process, donating electrons to HM ions through its surface oxygen-containing functional groups (e.g., -OH, Ar-OH, -COOH) and aromatic structures. This reduces HM ions to less toxic or insoluble lower-valence forms, thereby decreasing their mobility and bioavailability. A representative case is Cr^6+^ remediation: in soil, Cr^6+^ primarily exists as highly soluble and toxic chromate species (HCrO_4_^−^ and Cr_2_O_7_^2−^), as shown in Figure 11. Biochar’s oxygen-containing moieties (e.g., -OH, C=O, Ar-OH) act as localized electron reservoirs. These groups directly reduce Cr^6+^ to Cr^3+^ via single-electron transfer (Equations (2)–(5)) [87,92,93,94]:

(2) $$Cr{O_{4}}^{2-}+8H^{+}+3e^{-}\left(\text{-}NH_{2}\right)⇄Cr^{3+}+\text{-}NH\left(OH\right)+H_{2}O$$

(3) $$R\text{-}CH_{2}\text{-}OH\to R\text{-}\left(C=O\right)H+e^{-}$$

(4) $$\text{-}C\text{-}H+Cr^{6+}+H_{2}O⇄\text{-}C\text{-}OH+Cr^{3+}+H^{+}$$

(5) $$\text{-}C=O+Cr^{6+}+H_{2}O⇄\text{-}COOH+Cr^{3+}+H^{+}$$

**Figure 12:**
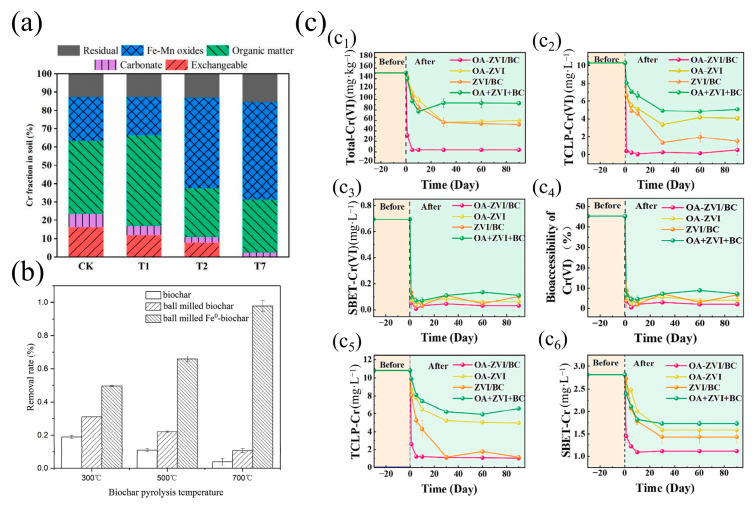
(**a**) Effect of compound type on the distribution of chromium species in soil [95]. (**b**) Removal rates of Cr^6+^ by 300BC, 500BC, 700BC, 300BMBC, 500BMBC, 700BMBC, 300BMFe^0^-BC, 500BMFe^0^-BC, and 700BMFe^0^-BC [96]. (**c**) Cr^6+^ and total Cr concentration in three extractions after the remediation of Cr^6+^ contaminated soil [97].

To enhance the reduction capacity of biochar and its immobilization efficiency for Cr^6+^, biochar needs to be modified. Fe-based materials are widely used due to their excellent performance (Figure 11). Yang et al. [95] modified ramie-derived biochar using nZVI, resulting in a fixation efficiency 3.27 times higher than that of the raw biochar, as shown in Figure 12a. This significant enhancement is partly attributed to the presence of the electron donor Fe^0^, which activates the oxygen-containing functional groups in the biochar, thereby improving its reduction capacity [38]. This further promotes the reduction and adsorption of Cr^6+^, as illustrated by Equations (6) and (7) [87]:

(6) $$Fe^{0}\to Fe^{2+}+2e^{-}$$

(7) $$3Fe^{2+}+HCr{O_{4}}^{-}+7H^{+}\to 3Fe^{3+}+Cr^{3+}+4H_{2}O$$

Surface modification strategies, particularly mechanochemical and acid-based approaches, have emerged as pivotal techniques for amplifying biochar’s capacity to immobilize Cr^6+^ through the synergistic optimization of its electron-donating architecture. In mechanochemically modified systems, Wang et al. [96] engineered Fe^0^-biochar composites via ball milling, achieving a 97.8% Cr^6+^ removal efficiency—a sixfold enhancement over pristine biochar (15.4%) (Figure 12b). This improvement arises from two interlinked mechanisms: (1) particle size reduction and increase in SSA, which exposes redox-active moieties (e.g., C=O and Ar-OH), and (2) in situ formation of Fe^0^/Fe_3_C heterojunctions during ball milling, which serve as electron pathways. These heterojunctions enable sustained electron donation to reduce Cr^6+^ while simultaneously suppressing Fe^3+^ passivation through carbide stabilization. In an acid-modified system, Xie et al. [97] demonstrated that oxalic acid (OA)-functionalized Fe^0^-biochar (OA-ZVI/B) achieved 96.7% long-term Cr^6+^ immobilization in soil (Figure 12c). OA serves dual roles as both a structural modifier and an electronic relay: its -COOH groups coordinate with Fe^0^ surfaces to form Fe–OOCR complexes, while the conjugated π-system of OA bridges electron transfer from Fe^0^ to Cr^6+^, facilitating direct solid-phase reduction. The resultant Cr^3+^ precipitates as Cr(OH)_3_ or forms complexes with biochar’s carboxyl sites, ensuring minimal remobilization.

### 3.7. Biochar in Synergistic Bioremediation

During the remediation of HM-contaminated soil, a single method often proves insufficient. Consequently, researchers in recent years have investigated combining biochar with other approaches to achieve more effective and stable results.

#### 3.7.1. Biochar in Combination with Plants

Remediating HM-contaminated soil presents significant challenges, including high HM concentrations [98] and the coexistence of multiple pollutants [99]. A single remediation technology is often insufficient for effective treatment, necessitating the integration of multiple approaches. Phytoremediation, recognized for its eco-friendliness and cost-effectiveness, has been widely employed in soil remediation. Studies have demonstrated that the combined application of biochar with plants such as ryegrass [98], castor seedlings [100], and Angelica [100] can significantly enhance HM stabilization in soil. This enhancement is primarily attributed to biochar’s dual function: directly immobilizing HMs while simultaneously improving soil properties to support plant growth, thereby increasing the plant’s capacity for HM accumulation. Additionally, biochar influences the distribution and translocation of HMs within plants, further improving remediation efficiency [101]. To optimize biochar-assisted phytoremediation, the selection of plant species should prioritize their tolerance to HMs, followed by considerations such as the life cycle, geographic distribution, root structure, biomass yield, and adaptability to specific soil pH conditions (acidic or alkaline) [102]. In this context, plant functional types play distinct roles: hyperaccumulators (e.g., *Brassica juncea*, *Sedum alfredii*) are commonly used for phytoextraction, while crops (e.g., maize, ryegrass) contribute to phytostabilization. Moreover, root exudates—particularly organic acids such as citric, oxalic, and malic acids—can chelate heavy metals, increasing their mobility. At the same time, these exudates facilitate microbial transformation and buffer rhizosphere pH, further enhancing the overall remediation process.

Currently, this combined remediation approach is being applied to the practical remediation of contaminated soil. For instance, Jun, Wei, Aili, Juan, Hongyan, Jingsong, Yunhua, and Cuiying [101] conducted a field experiment in the Shuikoushan mining area, Hengyang City, Hunan Province, using lychee branch biochar and sunflowers for joint remediation. The results demonstrated that applying biochar promoted sunflower growth and significantly enhanced its remediation effect on HMs. Compared to the control group, the total accumulation of Pb, Cd, and As in sunflower plants increased by 15.8–110% following the application of biochar. However, challenges remain in biochar and plant-based remediation: (1) The combinations of biochar and plants are highly variable, and systematic scientific methods for elucidating their mechanisms are lacking; (2) Specific technical guidelines are required to select suitable biochar–plant combinations tailored to local conditions for field implementation. Additionally, farmers need to consider the economic value of the plants being cultivated; (3) Ensuring that HMs absorbed by plants do not re-enter the soil environment is another critical issue.

#### 3.7.2. Biochar in Combination with Microorganisms

Similar to phytoremediation, the application of microorganisms in remediating HM-contaminated soils necessitates careful consideration of their tolerance to HMs. The primary goal of integrating biochar with microorganisms is to enhance their resistance and remediation capacity for HMs [103]. This strategy relies on biochar serving as a microbial carrier, offering a more stable growth environment and improving microbial adaptability to polluted conditions [104]. By creating favorable conditions for microbial activity, biochar facilitates the degradation and transformation of HMs through bioadsorption, bioaccumulation, and biotransformation. The microorganisms commonly employed in this process are primarily bacteria, including phosphate-solubilizing bacteria [105], *Pseudomonas* [106], *Bacillus* [107], and *Enterobacter* [104]. In contrast, fungi and algae are less frequently used in HM remediation due to their slower growth rates, the stringent cultivation requirements of fungi, and the specific aquatic conditions necessary for algae. However, certain fungal groups, particularly arbuscular mycorrhizal fungi (AMF), play a critical role in synergistic phytoremediation systems. AMF can enhance plant tolerance to heavy metals, improve nutrient uptake, and facilitate root–soil interactions, thereby complementing the effects of biochar and contributing to overall remediation efficiency. Although their integration into biochar-assisted strategies remains underexplored, future studies should consider their potential in multi-component remediation frameworks. Additionally, the introduction of exogenous microorganisms in biochar-assisted remediation may disrupt the native microbial community due to the competition between indigenous and introduced strains [107]. Therefore, practical applications must carefully balance the synergistic effects of microbial inoculation and biochar while mitigating potential ecological disturbances. Beyond microbial inoculation strategies, specific microbial consortia also contribute to redox-mediated transformations, and sulfur- and iron-reducing bacteria are commonly involved in biochar–microbe systems, facilitating redox transformations of Cr, As, and Pb. While biochar enhances microbial colonization and activity, low-molecular-weight organics from biochar or root exudates may also increase HM mobility. Therefore, the overall remediation effect depends on the balance between immobilization and potential mobilization under site-specific conditions.

## 4. Advantages of Biochar in Remediating HM-Contaminated Soil

### 4.1. Carbon Sequestration and Emission Reduction

The application of biochar in soil remediation not only improves soil quality but also provides significant carbon sequestration and emission reduction benefits. As a stable carbon reservoir, biochar increases soil carbon stocks, thereby contributing to long-term carbon storage. Its carbon structure is highly stable, enabling it to persist in the soil for centuries or even millennia without being readily decomposed by microorganisms, making it an effective long-term carbon sink. Additionally, biochar application can reduce the need for chemical fertilizers, indirectly lowering the energy consumption and greenhouse gas emissions associated with fertilizer production. By influencing the soil’s redox potential and microbial activity, biochar also helps mitigate the emissions of CH_4_ and N_2_O, further contributing to climate change mitigation. Beyond its role in carbon sequestration, biochar’s organic carbon content enhances soil nutrient and moisture retention, particularly for key nutrients such as potassium, phosphorus, and nitrogen. This improves plant growth and crop yields, which, in turn, further increases soil carbon storage, emphasizing the dual benefits of biochar in carbon sequestration and emission reduction [108].

### 4.2. Enhancing Soil Properties and Mitigating Erosion

The strategic application of biochar has emerged as a multifunctional solution for addressing HM contamination while concurrently enhancing agricultural productivity. The key point is that biochar orchestrates a tripartite remediation mechanism: (1) Direct immobilization via pH-driven precipitation and surface sorption. (2) Structural optimization through pore-mediated aggregation and hydraulic regulation. (3) Biological activation of microbial–plant synergies for long-term metal stabilization. Central to its mechanism is its capacity to modulate soil pH dynamics. When incorporated into acidic soils, biochar’s inherent alkalinity (pH = 8–12) neutralizes soil acidity, elevating pH levels [109]. This pH elevation induces the hydroxylation and carbonate precipitation of cationic HMs (e.g., Cd^2+^, Pb^2+^), effectively reducing their solubility and mobility through shifts in speciation equilibrium. Beyond pH regulation, biochar fundamentally reshapes soil physicochemical functionality. Its high surface charge density and oxygen-containing functional groups (e.g., Ar-OH, -COOH) enhance CEC by 20–50%, creating persistent binding sites for HM immobilization via ion exchange and surface complexation [110]. Concurrently, biochar improves electrical conductivity by 20% through the release of dissolved ions (K^+^, Ca^2+^, Mg^2+^) [110], synergistically enhancing nutrient retention while suppressing metal bioavailability.

Structurally, biochar’s hierarchical pore network (micro- and mesopores: 0.5–50 nm) and expansive SSA (100–800 m^2^∙g^−1^) drive soil physical restructuring [111]. These properties promote particle aggregation, increasing the formation of stable macroaggregates (>250 μm) and elevating soil porosity by 59–66% [110]. The resultant improvements in hydraulic conductivity and aeration mitigate waterlogging and erosion risks while optimizing root-zone gas exchange—critical conditions for plant establishment in contaminated soils. This leads to a mutually reinforcing cycle where biochar enhances soil quality and healthy plants further strengthen the soil structure. Moreover, biochar’s strong adsorption capacity enables it to adsorb organic molecules in the soil, improving conditions, which supports the proliferation of beneficial microorganisms. These microorganisms, by decomposing organic matter and cycling nutrients, improve soil health and further stabilize the soil structure.

To enhance field applicability, biochar design should consider site-specific conditions such as soil pH, contaminant speciation, and coexisting stressors. For acidic mine soils with polymetallic contamination, high-temperature biochars derived from manure or sludge may offer greater immobilization potential through mineral-driven precipitation and pH buffering. In contrast, for neutral agricultural soils with moderate HM levels, lignocellulosic biochars produced at lower temperatures may provide sufficient adsorption capacity while preserving the soil structure and microbial compatibility. Modification strategies (e.g., Fe/Mn oxides and thiol groups) can be selectively applied based on dominant metal species and redox conditions. These scenario-specific considerations may guide future material selection and optimization.

## 5. Conclusions and Outlook

Over the past decade, biochar has garnered considerable attention as an effective material for remediating soils contaminated with HMs. This review synthesizes 111 studies (2013–2025) to elucidate the primary mechanisms by which biochar facilitates HM immobilization. Key direct mechanisms include electrostatic adsorption, ion exchange, co-precipitation, surface complexation, and redox reactions (e.g., the reduction of Cr^6+^ to Cr^3+^ and the oxidation of mobile As^3+^). Particular attention is given to two understudied aspects: (1) biochar-induced soil pH elevation, which promotes the hydroxide precipitation of Cu^2+^ and Pb^2+^, and (2) emerging functionalization strategies, such as sulfur impregnation (enhancing Cd adsorption by 43.3%) and Fe-Mn oxide coatings (tripling Cu^2+^/Cd^2+^ removal efficiency). Beyond direct metal fixation, biochar indirectly contributes to soil remediation through carbon sequestration, improved soil physicochemical properties (e.g., enhanced porosity and CEC), and reduced erosion-driven metal mobility. This review underscores biochar’s dual role as a sustainable remediation agent, simultaneously immobilizing metals, enhancing soil health, and mitigating carbon emissions, positioning it as a cost-effective alternative to conventional technologies.

Although biochar demonstrates considerable potential for HM pollution remediation, its standalone application remains limited in certain scenarios and may not fully meet the demands of practical remediation efforts. To address these limitations, the integration of biochar with phytoremediation and microbial remediation has gained prominence as an effective strategy. Future research should explore engineered biochars with targeted functional groups, such as thiol (-SH), amino (-NH_2_), or phosphate (-PO_4_^3−^), which are designed to selectively bind specific metal mixtures (for example, Cd^2+^/Pb^2+^ or As^3+^/Cr^6+^). This approach can enhance remediation precision and efficiency. In addition, the development of multi-functional biochars that possess capabilities for simultaneous adsorption, redox transformation, and microbial support may provide promising solutions for complex contaminated sites. Beyond material design, it is essential to facilitate the transition from laboratory studies to large-scale applications. Most studies reviewed in this manuscript were conducted under laboratory conditions, which offer valuable mechanistic insights but may not fully reflect the complexity of real environmental settings. Although a few field-scale trials have been reported, the large-scale implementation of biochar-based remediation remains limited due to variability in soil properties, contaminant profiles, and concerns regarding long-term stability. Practical application in field conditions involves multiple considerations, including feedstock availability, processing methods, transportation logistics, and deployment strategies. These factors collectively influence the feasibility, scalability, and sustainability of biochar-based technologies. To validate the environmental and economic claims associated with biochar, long-term field trials and life-cycle assessment (LCA) studies are urgently needed. Future research should aim to bridge the gap between laboratory findings and real-world applications by addressing operational challenges, evaluating long-term performance, and developing context-specific implementation frameworks. In parallel, future studies should also assess potential secondary risks, such as contaminant desorption, nanoparticle release, and ecotoxic impacts, to ensure long-term environmental safety.

## Figures and Tables

**Table 1:** Physical and chemical properties of biochar.

Raw Material	Temperature (°C)	Time (h)	Rate (°C·min^−1^)	N%	C%	pH Value	CEC (cmol·kg^−1^)	C/H	C/N	Ash (%)	SSA (m^2^·g^−1^)	Average Pore Size (nm)	Ref
Cow manure	300	1	20	2.72	38.97	8.62	185.89 ^a^	0.93	/	34.54	3.42 ^c^	4.44	[20]
Cow manure	700	1	20	1.54	34.76	10.83	147.52 ^a^	3.44	/	50.45	10.61 ^c^	16.1	[20]
Orange peel	500	/	/	2.10	66	8.80	68.28 ^a^	1.52	36.66	11	0.21 ^c^	/	[21]
Sugar cane bagasse	500	/	/	1.00	74	9.60	69.60 ^a^	2.37	86.33	12	92.30 ^c^	65	[21]
Peanut hull	600	2	/	0.94	86.39	6.90	/	/	91.90	/	27.10 ^c^	/	[22]
Walnut shells	200	4	15	0.30	61.94	6.47	/	0.95	240.8	1.41	1.73 ^c^	/	[23]
Walnut shells	600	4	15	0.30	90.56	10.33	/	3.57	352.2	1.57	315.1 ^c^	/	[23]
Date palm leaf	600	2	5	0	76.23	10.23	39.86 ^b^	6.35	/	38.68	164.7 ^c^	4.92	[24]
Bamboo	600	2	/	0.15	80.89	7.9	/	/	539.3	/	470.4 ^c^	/	[22]
Corn	350	3	5	1.64	57.01	8.17	31.95 ^a^	1.22	34.72	28.13	32.1 ^c^	/	[25]
Corn	500	3	5	1.56	59.91	9.19	23.87 ^a^	2.32	38.49	33.00	61.83 ^c^	/	[25]
Rice straws	200	4	15	0.98	59.99	6.81	/	0.88	71.42	13.97	2.37 ^c^	/	[23]
Rice straws	600	4	15	1.67	90.79	12.39	/	3.13	63.43	27.22	211.9 ^c^	/	[23]

^a^: The CEC of biochar samples was measured using the NH_4_OAc method. ^b^: The CEC of biochar samples was measured using the NH_4_^+^-Na^+^ method. ^c^: SSA values were determined via N_2_-BET adsorption.

**Table 2:** Impact of biochar modification on SSA, pore structure, and HM immobilization efficiency.

Raw Material	Pyrolysis Temperature (°C)	Modification Method	SSA Before Modification (m^2^·g^−1^)	SSA After Modification (m^2^·g^−1^)	Pore Size Before Modification (nm)	Pore Size After Modification (nm)	Fixed HMs	Fixed Effect Before Modification	Fixed Effect After Modification	Ref
Poplar bark	600	SC(NH_2_)_2_ impregnation	2.77 ^c^	5.70 ^c^	1.94	6.42	Cd^2+^	36.26%	79.56%	[46]
Corncob	600	H_2_O_2_ modification	80.14 ^c^	196.4 ^c^	/	/	Pb^2+^	35.90%	39.86%	[47]
Rice straw	500	KOH modification	19.96 ^c^	54.66 ^c^	9.24	4.63	Zn^2+^	22.54 mg·g^−1^	25.37 mg·g^−1^	[45]
Corn stalks	600	KMnO_4_ modification	61.0 ^c^	2.28 ^c^	23.70	92.20	Cu^2+^	/	160 mg·g^−1^	[44]
Rice straw	500	HCl-HF modification	19.96 ^c^	22.45 ^c^	9.24	9.12	Zn^2+^	22.54 mg·g^−1^	16.53 mg·g^−1^	[45]
Rice straw	500	R-SH modification	7.82 ^c^	0.34 ^c^	20.82	16.69	Cd^2+^	41.20 mg·g^−1^	45.10 mg·g^−1^	[8]

^c^: SSA values were determined via N_2_-BET adsorption.

**Table 3:** Examples of precipitation immobilization of HM ions by modified biochar composites.

Raw Material	Temperature (°C)	Modification Method	Soil pH Value	Precipitation	Fixation Effect	Ref
Corn stalks	900	Nitrogen doped (urea)	/	CaC_2_O_4_, CaCO_3_	After modification, the adsorption capacity of Cd^2+^ by biochar increased from 55.87 mg·g^−1^ to 74.37 mg·g^−1^	[56]
Corn stalks	500	Zinc iron oxide	5.69	CdZnFe_2_O_4_	After modification, the content of extractable Cd in soil decreased by 12.77–57.45%	[57]
			7.87		In alkaline soil, DTPA-Cd content decreased by 23.73–52.50%	[57]
Wheat straw	500	MgO	7.4	Cd(OH)_2_, CdCO_3_	The extraction rate of Cd in soil decreased by 50.85%	[58]
*Oiltea camellia* shells	500	Na_2_SiO_3_	/	CdCO_3_, CdSiO_3_ and Cd_2_SiO_4_	After modification, the adsorption capacity of biochar for Cd^2+^ increased from 62.2 mg·g^−1^ to 120–211 mg·g^−1^	[59]
Corncob	600	MgO	9.14	Pb(OH)_2_, Pb_3_(CO_3_)_2_(OH)_2_	The extraction rate of Pb was reduced by 50.71%	[43]
Wood ear mushroom sticks	650	Calcium alginate	5.36	Ca_2.5_Pb_7.5_(OH)_2_(PO_4_)_6_	The effective concentration of Pb was reduced by 31.9–78.6%	[60]
			7.46		The effective concentration of Pb was reduced by 26.4–62.3%	[60]
Bamboo	800	Iron, aluminum and magnesium metal oxides	2.88	As-Fe/Ca precipitate	Fixed more than 85% As	[61]

**Table 4:** Complexation of biochar organic functional groups with HMs.

Raw Material	Pyrolysis Temperature (°C)	HM	Complexing Functional Group	Biochar Fixation Effect	Ref
Rice straw	500	Pb	-OH	The fixation rate of Pb in paddy soil was 50.47%	[72]
Crayfish shell	300–700	Pb	Ester group (-COOR) and -OH	The DTPA-Pb content in acidic soil was reduced by 1.87–16.48%	[73]
Poultry litter	300–500	Zn	-COOH or -OH	Reduced the content of acid-soluble Cd in soil by 8~10%	[74]
Wheat straw	550	Zn	-COOH, C=O and Ar-OH	/	[71]
Wheat straw	700	Cd	-COOH and -OH	Reduced the content of available Cd in soil by 47.97–61.38%	[75]
Rice straw	400	Cd	-COOR, C=O and -OH	The content of Cd in the roots, stems, and leaves of lettuce was reduced by 62–67%	[76]
Poultry litter	300–500	Cu	-COOH, Ar-OH, -OH and C=O	Reduced the acid-soluble Cd content in soil by 8–10%	[74]

## Data Availability

No new data were created or analyzed in this study.
